# High density genetic mapping of Fusarium head blight resistance QTL in tetraploid wheat

**DOI:** 10.1371/journal.pone.0204362

**Published:** 2018-10-11

**Authors:** Ehsan Sari, Samia Berraies, Ron E. Knox, Asheesh K. Singh, Yuefeng Ruan, Richard D. Cuthbert, Curtis J. Pozniak, Maria Antonia Henriquez, Santosh Kumar, Andrew J. Burt, Amidou N’Diaye, David J. Konkin, Adrian L. Cabral, Heather L. Campbell, Krystalee Wiebe, Janet Condie, Prabhath Lokuruge, Brad Meyer, George Fedak, Fran R. Clarke, John M. Clarke, Daryl J. Somers, Pierre R. Fobert

**Affiliations:** 1 Agriculture and Agri-Food Canada, Swift Current Research and Development Centre, Swift Current, Canada; 2 Aquatic and Crop Resource Development Centre, National Research Council Canada, Saskatoon, Canada; 3 Department of Plant Sciences, University of Saskatchewan, Saskatoon, Canada; 4 Morden Research and Development Centre, Agriculture and Agri-Food Canada, Morden, MB, Canada; 5 Brandon Research and Development Centre, Agriculture and Agri-Food Canada, Brandon, MB, Canada; 6 Ottawa Research and Development Centre, Agriculture and Agri-Food Canada, Ottawa, ON, Canada; 7 Vineland Research and Innovation Centre, Vineland, ON, Canada; Institute of Genetics and Developmental Biology Chinese Academy of Sciences, CHINA

## Abstract

Breeding for Fusarium head blight (FHB) resistance in durum wheat is complicated by the quantitative trait expression and narrow genetic diversity of available resources. High-density mapping of the FHB resistance quantitative trait loci (QTL), evaluation of their co-localization with plant height and maturity QTL and the interaction among the identified QTL are the objectives of this study. Two doubled haploid (DH) populations, one developed from crosses between *Triticum turgidum* ssp. *durum* lines DT707 and DT696 and the other between *T*. *turgidum* ssp. *durum* cv. Strongfield and *T*. *turgidum* ssp. *carthlicum* cv. Blackbird were genotyped using the 90K Infinium iSelect chip and evaluated phenotypically at multiple field FHB nurseries over years. A moderate broad-sense heritability indicated a genotype-by-environment interaction for the expression of FHB resistance in both populations. Resistance QTL were identified for the DT707 × DT696 population on chromosomes 1B, 2B, 5A (two loci) and 7A and for the Strongfield × Blackbird population on chromosomes 1A, 2A, 2B, 3A, 6A, 6B and 7B with the QTL on chromosome 1A and those on chromosome 5A being more consistently expressed over environments. FHB resistance co-located with plant height and maturity QTL on chromosome 5A and with a maturity QTL on chromosome 7A for the DT707 × DT696 population. Resistance also co-located with plant height QTL on chromosomes 2A and 3A and with maturity QTL on chromosomes 1A and 7B for the Strongfield × Blackbird population. Additive × additive interactions were identified, for example between the two FHB resistance QTL on chromosome 5A for the DT707 × DT696 population and the FHB resistance QTL on chromosomes 1A and 7B for the Strongfield × Blackbird population. Application of the Single Nucleotide Polymorphic (SNP) markers associated with FHB resistance QTL identified in this study will accelerate combining genes from the two populations.

## Introduction

Durum wheat (*Triticum turgidum* L. ssp. *durum* (Desf.) Husn.) is one of the major cereal food crops grown in the temperate regions of the world. Fusarium head blight (FHB) causes severe reductions in grain yield and quality in the growing regions with moist and warm weather. The impact on grain quality is in part through grain contamination with mycotoxins, which are harmful to human and animal health [1]. Integrated methods of control are practiced by producers, and FHB-resistant cultivars are an efficient and cost-effective component of the strategy to combat this disease. Different types of FHB resistance have been identified in hexaploid wheat [2]. Resistance to initial infection or incidence is known as Type I resistance and resistance to spread or severity is known as Type II resistance, both of which have been extensively studied in hexaploid wheat [2,3]. Type I and II resistance are also reported in the Canadian durum wheat line DT696 [4]. Using the available resistance in adapted sources such as line DT696 has the advantage of combining resistance in durum wheat cultivars with minimal detrimental effects of linkage drag, but such sources are rare within the breeding gene pool.

Resistance to FHB is reported from tetraploid species that are relatives of durum wheat such as *T*. *turgidum* ssp. *dicoccoides* [5], *T*. *turgidum* ssp. *dicoccum* [6,7] and *T*. *turgidum* ssp. *carthlicum* [7,8]. For example Some durum wheat landraces with moderate levels of FHB resistance have also been identified in Tunisian and Syrian germplasm [9,10]. The available levels of FHB resistance in locally adapted durum wheat lines could be boosted by introgression of resistance from non-adapted resistant sources. Linkage drag could be alleviated by identification of FHB resistance quantitative trait loci (QTL) from these more exotic sources, providing opportunity for recombination, and using the associated markers for marker-assisted backcrossing. The efficiency of marker-assisted backcrossing depends on population size and selection strategy as well as marker density and position [11]. High-density linkage maps could supply markers highly associated with the FHB resistance QTL allowing precise and efficient combining of the QTL from locally adapted and non-adapted germplasm for developing resistant durum wheat germplasm.

Genotyping technology has leapt forward during the past decade. The advent of next generation sequencing has supplied a large repository of Single Nucleotide Polymorphism (SNP) markers in wheat allowing high-density genotyping and QTL mapping [12,13]. High density genetic maps better identify and resolve QTL and SNP markers are preferred over other DNA markers, due to their abundance, high throughput and lower error rate [14]. Wang et al. [15] developed a high density SNP genotyping array containing 90,000 (90K) SNP markers largely based on RNA sequences of a diverse panel of tetraploid and hexaploid wheat lines. Using eight bi-parental mapping populations, they mapped 46,977 SNP markers. Later, Avni et al. [16] used the wheat 90K iSelect genotyping assay to produce an ultra-dense genetic map of durum wheat × *T*. *turgidum* ssp. *dicoccoides* containing 16,387 SNP markers. More recently, a consensus map for tetraploid wheat included 30,144 polymorphic SNP markers from the 90K iSelect genotyping assay by combining component maps of 13 bi-parental mapping populations derived from durum wheat, *T*. *turgidum* ssp. *dicoccum* and *T*. *turgidum* ssp. *dicoccoides* [17]. High-density linkage maps based on 90K iSelect markers are expected to improve the resolution of FHB resistance QTL analysis and enable identification of markers desirable for marker-assisted selection (MAS) and backcrossing.

Resistance to FHB is polygenic, hence requiring a quantitative approach to evaluation and analysis. QTL mapping of FHB resistance in tetraploid wheat has been previously conducted using bi-parental mapping populations [6,8,18–27]. Resistance QTL have been characterized on chromosomes 2A [27], 2B [8], 3A [19], 3B [6], 4A [25], 4B [20], 5A [21], 6B [8], 7A [6] and 7B [20]. Type II FHB resistance segregating in a doubled haploid (DH) population derived from the durum wheat cv. Strongfield × *T*. *turgidum* ssp. *carthlicum* cv. Blackbird population was previously evaluated in greenhouse trials and FHB resistance QTL on chromosomes 2B and 6B were identified [8]. Preliminary comparative mapping indicated that the FHB resistance QTL on chromosome 6B derived from cv. Blackbird co-located with the *Fhb2* locus [8], a major effect FHB resistance QTL present in the hexaploid wheat line Sumai 3 [28]. Simple Sequence Repeat (SSR) markers flanking these QTL were reported for use in marker-assisted selection; however, more highly associated SNP markers are more desirable for reducing linkage drag of cv. Blackbird. Phenotyping under field conditions is required to fully assess and validate greenhouse testing of cv. Blackbird FHB resistance in targeted durum wheat production environments.

FHB resistance is associated with a number of developmental traits such as plant height, flowering time and spike morphology [20]. Previous studies highlighted a negative correlation between plant height and FHB severity, taller lines being more resistant [18,29]. This is supported by the co-localization of FHB resistance with height QTL in previous studies [20,21,30]. For example, the association of a FHB resistance QTL with the plant height gene *Rht-B1* was suggested in a population derived from *T*. *turgidum* ssp. *dicoccum* × *T*. *turgidum* ssp. *durum* cv. Helidur [20]. The main spike traits that are associated with resistance to FHB include anther extrusion and anthesis date [21,31]. Buestmayr et al. [21] found a FHB resistance QTL on chromosome 5A from *T*. *macha* co-located with the *Q*-gene that controls plant height, and spike traits including anthesis date, and spike density and length. The association of FHB resistance with these traits requires evaluation and co-selection of several traits simultaneously while breeding for FHB resistance. Marker-assisted selection using SNP markers highly associated with the QTL mediating FHB resistance and desirable developmental traits should improve the throughput of breeding programs by identifying lines carrying favourable alleles in early generations e.g. the F_2_.

The cultivar Blackbird is a source of FHB resistance [8] and durum wheat line DT696 has been used as an adapted source of FHB resistance in the breeding program at the Swift Current Research and Development Centre (SCRDC) of Agriculture and Agri-Food Canada (AAFC). Durum wheat varieties showing some improvement in FHB resistance, such as Brigade, Transcend and CDC Credence, derived from line DT696 [32,33]. QTL mapping of FHB resistance in line DT696 and cv. Blackbird is necessary to identify markers for use in MAS. The objectives of this study were to identify and map FHB resistance QTL with high density genetic maps of DH populations developed from crosses of lines DT707 × DT696 and durum wheat cv. Strongfield × *T*. *turgidum* ssp. *carthlicum* cv. Blackbird, to investigate the co-localization of identified QTL with plant height and maturity and to identify the interactions between the identified QTL.

## Materials and methods

### Plant materials

Two DH populations were evaluated for FHB resistance in this study. The A0132& population was developed from a cross of advanced Canadian breeding lines DT707 (AC Avonlea/DT665) × DT696 (DT618/DT637//Kyle), and the A0022& population from a cross of durum wheat cv. Strongfield × *T*. *turgidum* ssp. *carthlicum* cv. Blackbird. DH lines were developed using the maize pollination technique [34]. Strongfield is a spring durum wheat cultivar adapted to the semi-arid environment of the northern Great Plains [35]. Lines DT696 and DT707, and cv. Strongfield were developed at the SCRDC.

### FHB, plant height and maturity phenotyping

The DH populations were evaluated for resistance to FHB in multiple field nurseries over years (S1 Table). The experiments were conducted as alpha-lattice field designs with 12 entries per incomplete block for the A0022& population and 17 for the A0132& population. Plots were 1 m long single rows with 50–100 seeds per row. Phenotyping was initiated with 121 lines of the A0132& population at the Carman, MB nursery in 2005, 2006 and 2007, and at thePortage la Prairie, MB nursery in 2006 and 2007, and 90 lines of the A0022& population at Carman and Portage la Prairie in 2006 and 2007. There were two replicates for field trials conducted in Carman and Portage la Prairie in 2005–2007. The population sizes were increased to 423 for the A0132& population and 102 lines for the A0022& for unreplicated trials conducted in Morden, Brandon and Indian Head in 2015–2017. Plant height and relative maturity data were collected from nine separate field trials in three locations for 121 lines of the A0132& population and six trials in two locations for 90 lines of the A0022& population (S1 Table). There were two replicates for field trials subjected to plant height and relative maturity measurements except for the unreplicated trials at Lethbridge in 2004 for the A0132& population and at Swift Current in 2014 for the A0022& population.

The FHB nurseries at Portage la Prairie, Brandon and Morden were inoculated with corn spawn colonized with a mixture of aggressive 3-acetyl-deoxynivalenol (3ADON) and 15-acetyl-deoxynivalenol (15ADON) producing strains of *Fusarium graminearum*. The Morden nursery was inoculated approximately 2–3 weeks prior to heading, while at the Brandon nursery the first application of corn inoculum was done at 6 weeks after planting followed by another application 2 weeks later after the first application. Colonized corn grains were broadcasted between the rows at the rate of 20 g m^-2^ at Portage la Prairie, 40 g m^-2^ at Brandon, and at 8 g per row, twice at weekly intervals at Morden. Nurseries at Portage la Prairie and Brandon were irrigated three times a week with an overhead low pressure mist irrigation system immediately upon completion of inoculation to maintain dew on the spikes for the disease development. Nurseries at Morden were irrigated three times a week using Cadman Irrigation travellers with Briggs booms. At the Carman nursery, the spikes of the plots were spray-inoculated using a CO_2_ pressurized backpack sprayer calibrated at 2 kPa at 50% anthesis with 50 ml per row conidia suspension composed of a mixture of aggressive 3ADON and 15ADON producing strains of *F*. *graminearum*. The concentration of the conidial suspensions was adjusted to 5 × 10^4^ conidia mL^-1^ using a hemocytometer and Tween 20 (1 drop per 100 ml) was added to the suspension. Inoculation was repeated 4 d after the first inoculation. The nursery was mist irrigated in the evening, and the morning after each inoculation. The FHB nursery at Indian Head was dependent on natural infection.

FHB incidence (percentage of spikes showing symptoms) and severity (percentage of spike area infected) were recorded for each plot. FHB index was calculated from the incidence and severity rating data using the formula (incidence × severity)/100. FHB intensity (visual rating of whole plot for infection using a 1 to 9 scale: 1: no infection and 9: ≥ 90% infection) was collected at Portage La Prairie in 2006 and 2007. Plant height was measured on a representative plant from the soil surface to the tip of spikes excluding the awns. Relative maturity was rated using a 1–6 scale (1 = earliest and 6 latest maturity) when 80% or more of the plots had yellow heads, by pinching the seeds and comparing their moisture levels with the parents.

### Genotyping and developing high-density linkage maps

Lines were genotyped using the wheat iSelect 90K SNP genotyping assay following the method described by Wang et al. [15]. The SNP clustering was performed in GenomeStudio software v. 2011.1 (Illumina Inc., San Diego, CA, U.S.A.) using the default clustering algorithm and following the workflow described by Cavanagh et al. [36].

Draft linkage maps were generated using the MSTMap software [37] with a cut off *P*-value of 1×10^−10^ and a maximum distance between markers of 15.0 cM for grouping SNP markers into linkage groups. Draft maps were refined using the MapDisto version 1.7.5 software [38]. A cut-off recombination value of 0.35 and threshold logarithm of odds (LOD) score of 3.0 was used for reconstruction of linkage groups. Distances (cM) between markers were calculated using the Kosambi mapping function [39]. The linkage groups were then checked individually for the presence of double recombinants and markers with double recombination events were re-scored. The order of markers was refined for inversion events using “Check Inversions” and “Auto Ripple” commands. The linkage groups were assigned to the wheat chromosomes based on existing high density SNP maps [15,17,36].

### QTL mapping

QTL interval mapping was conducted using MapQTL v. 5.0 [40]. A QTL interval was considered significant if the LOD score exceeded a genome-wide significant threshold level at *P* = 0.05, determined by a 1,000 permutation test [41]. Automatic co-factor detection based on backward elimination was used to select co-factor markers for Multiple QTL Mapping (MQM). MQM was performed with the selected markers and a marker with the highest LOD score within each QTL interval identified from interval mapping. The least square means of two replicates was used as quantitative data for QTL mapping of replicated experiments. Type II greenhouse FHB data generated by Somers et al. [8] for the 90 line subset of the A0022& population was used to remap the FHB resistance QTL on chromosomes 2B and 6B with the high density SNP marker map. To enable the presentation of all the QTL intervals on a single map, the location of QTL detected using the map generated for the small subsets of both populations were projected on the linkage map of bigger subsets by anchoring the shared markers. The QTL graphs were prepared using MapChart 2.2 [42] using LOD scores of MQM and the LOD threshold determined by the permutation test. The two-dimensional two QTL scan tool of R/qtl software [43] was used to identify the interacting loci and determine the type of their interactions.

### Statistical analysis

Variance analysis was performed on the replicated trials using Statistical Analysis System (SAS) software version 9.3 (SAS Institute Inc., Cary, NC, USA). Homogeneity of variances was tested using the Levene’s test and, in the case of heterogeneity, the variances were modelled using the SAS mixed model procedure. To estimate the variance due to line, the mixed model was used with line assigned as fixed effects and block nested in rep, year nested in location and line-by-year-by-location interaction assigned as a random effect. Means of lines were compared based on least significant differences with the Tukey adjustment (α = 0.05). Spearman’s rank correlation analysis was conducted for each of the FHB traits acquired at multiple locations in 2015–2017 using PROC CORR of SAS. Correlation was also conducted between the FHB ratings acquired at multiple locations in 2015–2017 and the plant height and relative maturity data acquired prior to 2015. The broad-sense heritability coefficient was estimated as a function of variance components according to the method suggested by Holland et al. [44]. For the estimation of the heritability coefficients, all effects were considered random.

## Results

### Trait variation, correlation and heritability

Variance analysis was conducted for all the replicated trials of FHB ratings, plant height and relative maturity. The line variation for the FHB traits, plant height and maturity of the A0132& population was highly significant (Table 1). It was highly significant for FHB index, plant height and maturity and significant for FHB incidence traits, but not significant for FHB severity of the A0022& population (Table 2). The difference between means of parents was significant for FHB incidence at Portage la Prairie in 2007 and for FHB severity and index at Portage la Prairie in 2006 and 2007 for the A0132& population where line DT696 had lower FHB symptoms than line DT707. The difference between means of parents was significant for FHB incidence, severity and index at Carman in 2006 and at Portage la Prairie in 2006 for the A0022& population. The cultivar Blackbird had lower FHB symptom than cv. Strongfield at Portage la Prairie in 2006 but had higher FHB symptom than cv. Strongfield at Carman in 2006. The heritability was low to moderate for FHB traits and moderate to high for plant height and maturity traits of both populations.

**Table 1:** Analysis of variance of line means, heritability (*H*), means of parents and population, and minimum and maximum values of Fusarium head blight (FHB), plant height and relative maturity traits from replicated field trials conducted for the DT707 × DT696 (A0132&) population.

Traits	Line *P* value^&^	*H*^$^	Environment (location/year)	μ _DT707_, μ _DT696_ (Significance of difference between means of parents)	Population		
					mean	max	min
FHB incidence	0.0001	0.43	CAR†*2005	60, 35.5 (ns)	42.4	85	10
			CAR2006	47.5, 25 (ns)	34	90	5
			CAR2007	75, 75 (ns)	76	100	30
			PLP#2006	70, 45 (ns)	66.5	100	10
			PLP2007	100, 85 (**)	98.2	100	80
FHB severity	0.0022	0.39	CAR2005	47.5, 27.5 (ns)	39.4	95	10
			CAR2006	12.5, 15 (ns)	32	90	5
			CAR2007	72.5, 47.5 (ns)	61	100	25
			PLP2006	55, 20 (**)	26.9	60	10
			PLP2007	70, 25 (**)	44.4	80	15
FHB index	< .0001	0.68	CAR2005	29.8, 10.7 (ns)	17.6	63.7	1
			CAR2006	7, 3.8 (ns)	11.2	56	0.3
			CAR2007	54.1, 37.1 (ns)	47.7	100	7.5
			PLP2006	39.5, 9 (**)	18.9	60	1
			PLP2007	70, 21.5 (**)	43.9	80	12
Plant Height	< .0001	0.70	Reg†2005	80, 100 (**)	92	110	50
			Reg2006	80, 80 (ns)	71	90	50
			Reg2007	69, 89.5 (**)	77.6	90	64
			SC††2005	80, 110 (**)	88	110	70
			SC2006	86, 84 (ns)	84.8	86	72
			SC2007	70, 70(ns)	69.7	80	60
Maturity	< .0001	0.86	Reg2005	3.5, 4 (ns)	4.1	6	2
			Reg2006	4.5, 6 (**)	4.9	6	3
			Reg2007	3.5, 4 (ns)	3.8	6	3
			SC††2005	3.5, 4 (ns)	3.9	5	3
			SC2006	3.5, 5 (**)	4.4	6	3
			SC2007	4, 5.5 (**)	4.5	6	3

The experiments were conducted as alpha-lattice designs with two replicates.

^&^*P* values of line variance estimated by mixed model when line was assigned as fixed effects, block nested in rep, year nested in location and line-by-year-by-location interaction assigned as a random effect.

^$^ Coefficient of broad-sense heritability estimated as function of variance components. Means of lines were compared based on least significant differences with the Tukey adjustment (α = 0.05).

Significance of difference among means of parents (μ) was denoted with * for *P* ≤ 0.05 and ** for *P* ≤ 0.01.

FHB incidence is percentage of spikes showing symptoms and severity is percentage of spike area infected. FHB index was calculated from the incidence and severity rating data using the formula (incidence × severity)/100. Plant height was measured on a representative plant from the soil surface to the tip of the spike excluding the awns. Maturity was rated using a 1–6 scale (1 = earliest and 6 latest maturity) when 80% or more of the plots had yellow spikes. The trial locations were.

#Portage la Prairie

†*Carman

†Regina and

††Swift Current

**Table 2:** Analysis of variance of line means, heritability (*H*), means of parents and population, and minimum and maximum values of Fusarium head blight (FHB), plant height and relative maturity traits from replicated field trials conducted for the Strongfield × Blackbird (A0022&) population.

Traits	Line *P* value^&^	*H*^*$*^	Environment (location/year)	μ _Strongfield_, μ _Blackbird_ (Significance of difference between means of parents)	Population		
					mean	max	min
FHB incidence	0.0297	0.41	CAR†*2006	50, 95 (**)	66.1	100	5
			CAR2007	82.5, 80 (ns)	88.5	100	40
			PLP#2006	70, 10 (**)	49.8	100	10
			PLP2007	100, 100 (ns)	96.5	100	60
FHB severity	0.6428	0.56	CAR2006	50, 90 (*)	61.1	95	5
			CAR2007	80, 77.5 (ns)	79.1	100	30
			PLP2006	30, 10 (**)	28.7	90	10
			PLP2007	30, 25 (ns)	37.1	70	15
FHB index	0.0009	0.53	CAR2006	25.5, 85.5 (**)	42.2	90.2	0.3
			CAR2007	66, 62.1 (ns)	70.6	100	12
			PLP2006	21, 1 (**)	18.5	90	1
			PLP2007	30, 25 (ns)	36.4	100	12
Plant height	< .0001	0.66	Reg†2005	82.5, 97.5 (ns)	81.2	105	60
			Reg2006	80, 90 (ns)	82	120	60
			SC††2005	90, 100 (*)	96	130	60
			SC2006	83, 87 (ns)	83.1	96	64
			SC2012	97, 105 (*)	102	120	80
Maturity	< .0001	0.72	Reg2005	4, 3 (*)	3.8	6	2
			Reg2006	4, 4.2 (ns)	4.5	6	3
			SC2005	3, 4.5 (*)	4.2	6	3
			SC2006	3, 3 (ns)	3.3	5	3
			SC2012	3.5, 5 (**)	4.2	6	3

The experiments were conducted as alpha-lattice designs with two replicates.

^&^*P* values of line variance estimated by mixed model when line was assigned as fixed effects, block nested in rep, year nested in location and line-by-year-by-location interaction assigned as a random effect.

^$^ Coefficient of broad-sense heritability estimated as function of variance components. Means of lines were compared based on least significant differences with the Tukey adjustment (α = 0.05).

Significance of difference among means of parents (μ) was denoted with * for *P* ≤ 0.05 and ** for *P* ≤ 0.01. FHB incidence is percentage of spikes showing symptoms and severity is percentage of spike area infected.

FHB index was calculated from the incidence and severity rating data using the formula (incidence × severity)/100. Plant height was measured on a representative plant from the soil surface to the tip of the spike excluding the awns. Maturity was rated using a 1–6 scale (1 = earliest and 6 latest maturity) when 80% or more of the plots had yellow spikes. The trial locations were

#Portage la Prairie

†*Carman

†Regina and

††Swift Current

The result of correlation analysis for the A0132& population is summarized in Table 3 and of the A0022& population in Table 4. Generally, correlations were low but rarely non-significant with FHB traits measured over environments and between FHB traits and plant height, and FHB traits and maturity, although some correlations were moderate for the A0022& population. Correlations of FHB with plant height and maturity traits were negative, reflecting lower FHB symptoms on tall and later maturing lines.

**Table 3:** Spearman’s rank correlation between each of the Fusarium head blight (FHB) traits (incidence, severity and index) measured over environments during 2015–2017, and between the FHB traits and, plant height and relative maturity data for the DT707 × DT696 (A0132&) population.

Trait	Environment	FHB incidence		
		MD2015	MD2016	MD2017
FHB Incidence	MD#2015		ns§	0.26**
	MD2016			0.25**
	MD2017			
Plant height	Reg†2007	ns	ns	-0.19*
Maturity	SC††2005	ns	ns	-0.21*
		FHB severity		
		MD2015	MD2016	MD2017
FHB severity	MD2015		0.25**	0.23*
	MD2016			0.27**
	MD2017			
Plant height	Reg2007	-0.21*	-0.25**	-0.23*
Maturity	SC2005	-0.24**	-0.22*	-0.22*
		FHB index		
		MD2015	MD2016	MD2017
FHB index	MD2015		0.25**	0.28**
	MD2016			0.28**
	MD2017			
Plant height	Reg2007	-0.20*	-0.23*	-0.23**
Maturity	SC2005	-0.19*	-0.17*	-0.22*

Only the coefficient of correlation (*r*) of significant correlations (*P* < 0.05) are reported and significance levels are denoted by ‘*’ for *P* < 0.05, and ‘**’ for *P* < 0.01.

The plant height and relative maturity data used for correlation analysis were measured prior to 2015. Only the results of correlation analysis for plant height and relative maturity data of a single trial with the highest correlation coefficient with the FHB rating are presented. The trial locations were

#Morden

†Regina

††Swift Current.

§ Non-significant *P* value (*P* > 0.05)

**Table 4:** Spearman’s rank correlation between each of the Fusarium head blight (FHB) traits (incidence, severity and index) measured over environments during 2015–2017, and between the FHB traits and, plant height and relative maturity data for the Strongfield × Blackbird (A0022&) population.

		FHB incidence					
Trait	Environment	MD2015	IH2015	MD2016	BD2016	MD2017	BD2017
FHB Incidence	MD#2015		0.22*	0.33**	0.42**	0.36**	0.37**
	IH$2015			0.22*	0.27**	ns§	0.22*
	MD2016				0.35**	0.22*	0.35**
	BD*†2016					0.19*	0.60**
	MD2017						0.23*
	BD2017						
Plant height	SC††2012	-0.33**	ns	-0.21**	-0.28**	-0.35**	-0.25*
Maturity	Reg†2006	ns	ns	ns	-0.18*	ns	ns
		FHB severity					
		MD2015	IH2015	MD2016	BD2016	MD2017	BD2017
FHB severity	MD2015		0.19*	0.21*	0.43**	0.31**	0.46**
	IH2015			0.25**	0.19*	0.41**	0.21*
	MD2016				0.31**	0.26**	0.35**
	BD2016					0.22*	0.36**
	MD2017						0.24*
	BD2017						
Plant height	SC2012	-0.17*	-0.22*	-0.25**	-0.24*	-0.33**	-0.18*
Maturity	Reg2006	ns	-0.27**	-0.23*	-0.26*	-0.21*	-0.19*
		FHB index					
		MD2015	IH2015	MD2016	BD2016	MD2017	BD2017
FHB index	MD2015		0.25**	0.27**	0.51**	0.34**	0.48**
	IH2015			0.29**	0.22*	0.32**	0.23*
	MD2016				0.33**	0.22*	0.29**
	BD2016					0.24*	0.49**
	MD2017						0.27**
	BD2017						
Plant height	SC2012	-0.28**	-0.22*	-0.23*	-0.26*	-0.40**	-0.27*
Maturity	Reg2006	ns	-0.23*	ns	-0.26*	ns	-0.23*

Only the coefficient of correlation (*r*) of significant correlations (*P* < 0.05) are reported and significance levels are denoted by ‘*’ for *P* < 0.05, and ‘**’ for *P* < 0.01.

The plant height and relative maturity data used for correlation analysis were measured prior to 2015. Only the results of correlation analysis for plant height and relative maturity data of a single trial with the highest correlation coefficient with the FHB rating are presented. The trial locations were

#Morden

†Regina

††Swift Current

$Indian Head and

*†Brandon.

§ Non-significant *P* value (*P* > 0.05)

### Linkage map and QTL analysis

The linkage map and results of QTL mapping for the A0132& population are provided in S1 File and for the A0022& population in S2 File. The map generated for the A0132& population consisted of 2,943 SNP markers in 19 linkage groups with an average marker density of 0.6 cM. The total length of the map was 1,808.4 cM. The map generated for the A0022& population consisted of 9,568 SNP markers in 15 linkage groups with an average marker density of 0.3 cM. The total length of the map was 2,762.9 cM.

**Fig 1:**
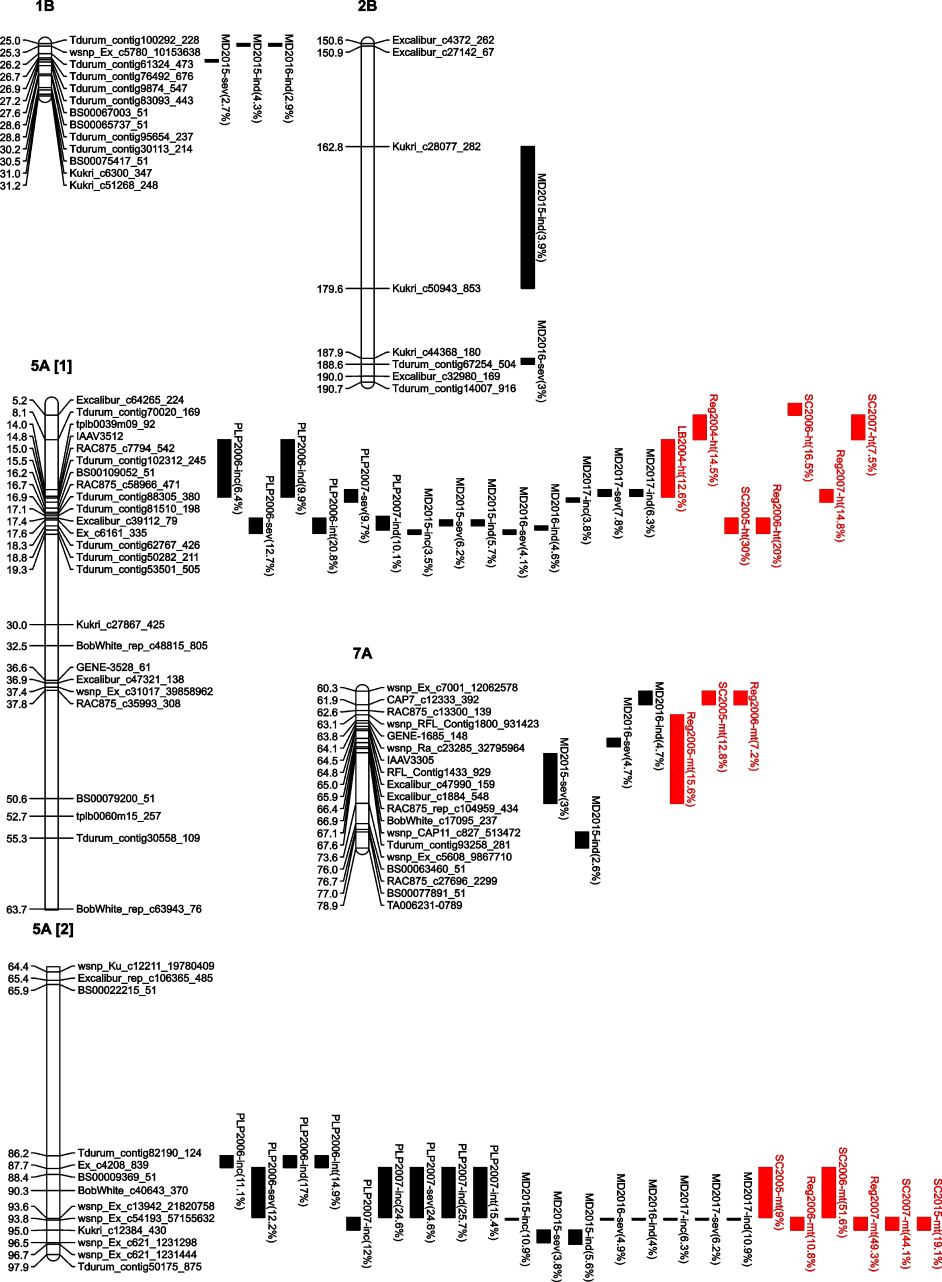
Chromosomal map position and percentage of phenotypic variation (in parenthesis) of Fusarium head blight (FHB) resistance QTL (black bars), and plant height and maturity QTL co-located with the FHB resistance QTL (red bars) identified for the DT707 × DT696 (A0132&) population. The traits subjected to QTL mapping were FHB incidence (inc), severity (sev), index (ind) and intensity (int) and plant height (ht) and relative maturity (mt). The trial locations were Portage la Prairie (PLP), Lethbridge (LB), Morden (MD), Regina (Reg), and Swift Current (SC). The results are from the Multiple QTL Mapping method using a LOD threshold based on a 1,000 permutation test at *P* = 0.05.

QTL mapping of FHB traits identified five QTL from the A0132& population on chromosomes 1B, 2B, 5A (two loci) and 7A (Fig 1). The resistance alleles belonged to line DT696 for all QTL (S1 File). The two FHB resistance QTL identified on chromosome 5A (5A1; spanning from 8.1–19.3 cM and 5A2 from 86.2 to 96.5 cM) were expressed fairly consistently among the environments. By contrast, FHB resistance QTL on chromosomes 1B, 2B and 7A were only identified for the data acquired in 2015–2017 using the large A0132& population (423 lines). The percentage of phenotypic variance explained by the QTL was generally lower for those on chromosomes 1B, 2B and 7A than on 5A. It ranged from 2.6 to 4.7% for QTL on chromosome 1B, 2B, and 7A, from 3.8 to 20.8% for 5A1 and from 3.8 to 25.7% for 5A2 QTL. The 5A1 QTL co-located with a plant height QTL while the 5A2 QTL and the QTL on chromosome 7A co-located with a maturity QTL. The tall and late maturity alleles derived from line DT696 (S1 File). QTL mapping also identified a plant height QTL on chromosome 6B and a maturity QTL on chromosome 7A that were not co-located with FHB resistance QTL (S1 File).

**Fig 2:**
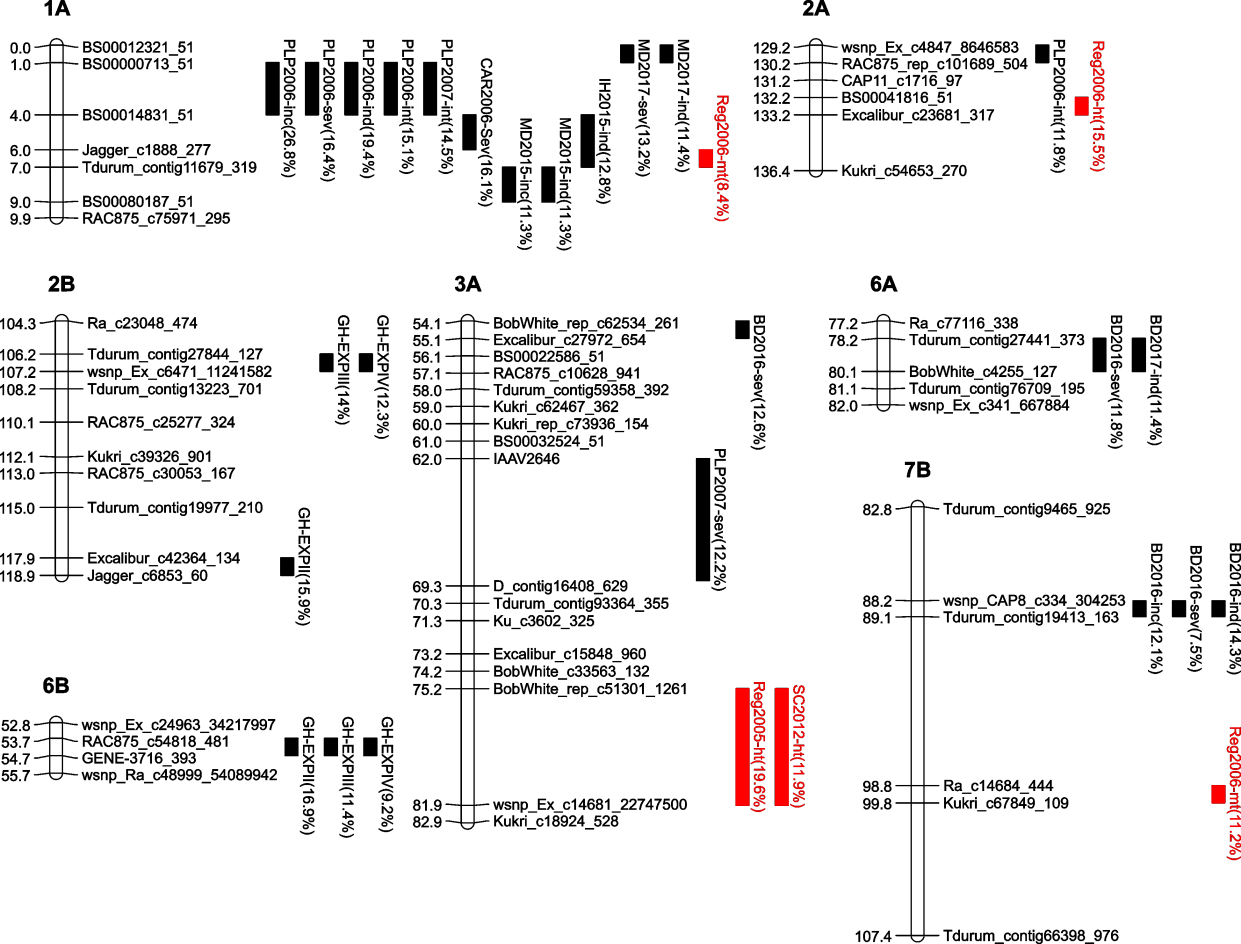
Chromosomal map position and percentage of phenotypic variation (in parenthesis) of Fusarium head blight (FHB) resistance QTL (black bars), and plant height and maturity QTL co-located with the FHB resistance QTL (red bars) identified for the Strongfield × Blackbird (A0022&) population. The traits subjected to QTL mapping were FHB incidence (inc), severity (sev), index (ind) and intensity (int), and plant height (ht) and relative maturity (mt). The trial locations were Portage la Prairie (PLP), Carman (CAR), Indian Head (IH), Morden (MD), Brandon (BD), Regina (Reg), and Swift Current (SC). The Type II FHB rating under greenhouse conditions from three independent studies (GH-EXPII, III, IV) were also analyzed. The results are from the Multiple QTL Mapping method using a LOD threshold based on a 1,000 permutation test at *P* = 0.05.

MQM analysis identified seven FHB resistance QTL from the A0022& population on chromosomes 1A, 2A, 2B, 3A, 6A, 6B and 7B (Fig 2). The resistance allele belonged to cv. Blackbird for the QTL on chromosomes 1A, 2A, 3A and 6B and to cv. Strongfield for the QTL on chromosomes 2B, 6A and 7B (S2 File). The FHB resistance QTL on chromosome 1A was expressed fairly consistently among the environments, but was not identified at the Brandon nursery. The FHB resistance QTL on chromosome 2A was only detected in one environment but co-located with a plant height QTL. The five other FHB resistance QTL were detected in a minimum of two locations. Previously Somers et al. [8] detected Type II FHB resistance QTL on chromosomes 2B and 6B using point inoculations in the greenhouse. Using these Type II data to remap QTL, we identified SNP markers associated with the FHB resistance QTL on chromosomes 2B and 6B. The percentage of phenotypic variance explained by the QTL on chromosome 1A ranged from 11.3 to 26.8%, on chromosome 2A was 11.8%, on chromosome 3A ranged from 12.2 to 12.6%, on chromosome 6A from 11.4 to 11.8% and on chromosome 7B from 7.5 to 14.3%. The FHB resistance QTL on chromosome 2A co-located with a plant height QTL and that on 3A located near a plant height QTL. The tall allele of the QTL on chromosomes 2A and 3A belonged to cv. Blackbird (S2 File). The FHB resistance QTL on chromosomes 1A and 7B co-located with a maturity QTL. The late maturity allele of the QTL on chromosome 1A belonged to cv. Blackbird and that on chromosome 7B to cv. Strongfield (S2 File). QTL mapping also identified plant height QTL on chromosomes 3B and 5A that were not associated with FHB resistance. The plant height QTL on chromosome 5A co-located with a maturity QTL (S2 File).

**Fig 3:**
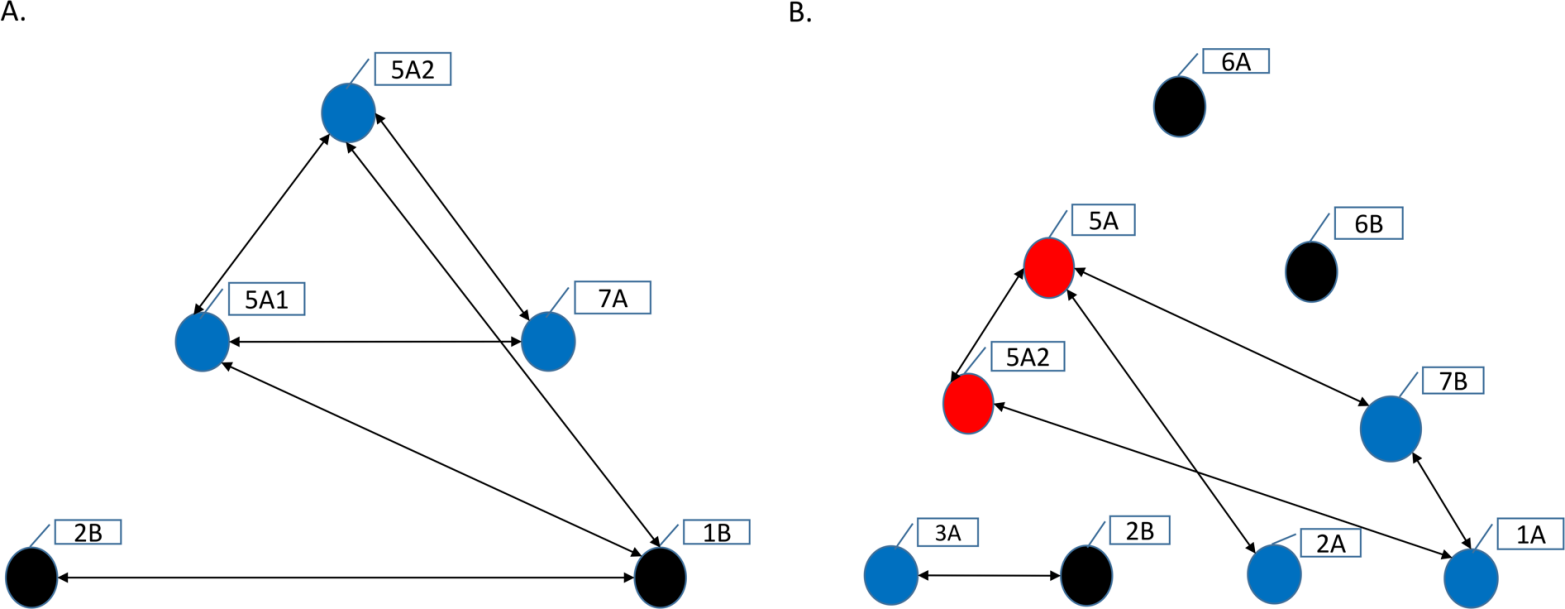
Interaction among Fusarium head blight (FHB) resistance QTL (black circles), FHB resistance QTL co-located with plant height and maturity QTL (blue circles) and other consistently interacting loci that were not detected as FHB resistance QTL (red circles). **A. interaction identified for the DT707 × DT696 (A0132&) population and B. for the Strongfield × Blackbird (A0022&) population.** The names of chromosomes with interacting QTL are denoted in boxes. Double arrows connecting the circles indicate additive × additive interactions.

The results of genome-wide QTL interaction analysis are summarized in S1 File for the A0132& and in S2 File for the A0022& population. Significant additive × additive interaction was detected between 5A1 and 5A2, 5A1 and 7A, 5A2 and 7A, 5A1 and 1B, and 1B and 2B FHB resistance QTL for the A0132& population (Fig 3A). Except for the FHB resistance QTL on chromosome 2B, all others interacted additively with either the 5A1 or 5A2 QTL.

The FHB resistance QTL on chromosome 1A had significant additive × additive interaction with that on chromosome 7B and the FHB resistance QTL on chromosome 2B had significant additive × additive interaction with that on chromosome 3A for the A0022& population (Fig 3B). The QTL on chromosome 1A also had significant additive × additive interaction with loci on chromosomes 4A, 4B and 7A, none of which were detected as significant FHB resistance QTL (S2 File). The FHB resistance QTL on chromosomes 2A and 7B interacted with the plant height QTL on chromosome 5A. No significant interaction was identified between the FHB resistance QTL on chromosomes 6A and 6B and the other QTL. A locus on chromosome 5A (marked as 5A2 in Fig 3B), which was not detected as a significant FHB resistance QTL, interacted consistently with the FHB resistance QTL on chromosome 1A and with the plant height QTL on chromosome 5A. Comparative mapping analysis using the SNP markers shared between the linkage maps of the A0022& and A0132& populations suggested that the interacting loci on chromosome 5A co-located with the 5A2 FHB resistance and maturity QTL detected for the A0132& population. In general, the interactions were more frequently detected between the FHB resistance QTL co-located with the plant height and maturity QTL than those solely associated with FHB resistance.

## Discussion

Genotype-by-environment interactions for the expression of FHB resistance were observed for the A0022& and A0132& populations. This was supported by the low to moderate heritability of FHB traits, low to moderate correlation of FHB traits and the inconsistent expression of QTL across environments. Some of the QTL including that on chromosomes 1A from the A0022& population and those on chromosome 5A from the A0132& population were more consistently expressed across environments, making them desirable candidates for utilization in breeding programs. Verges et al. [45] estimated a broad-sense heritability of 0.3 for FHB severity in a number of soft red winter wheat breeding populations under field conditions. The data used for estimating heritability in our study were from two nearby FHB nurseries in different years (Carman and Portage la Prairie, MB), one potential reason for higher heritability than that reported by Verges et al. [45]. In contrast, correlation was assessed using data of more distant locations over years. The correlation of FHB traits across environments was often low and occasionally moderate supporting the low heritability of FHB traits as suggested by Verges et al. [45]. Marker-assisted selection is recommended for traits with low heritability such as FHB resistance. Marker-assisted stacking of the available minor to moderate effect FHB resistance loci reported in this study seems a viable strategy for improving resistance to FHB in durum wheat.

The correlations of FHB traits with plant height and maturity suggest an association of these traits with resistance to FHB for both the A0022& and A0132& populations. Previous studies also inferred the association of developmental traits with the expression of FHB resistance [20,46]. Furthermore, QTL mapping identified FHB resistance QTL co-located with plant height and maturity QTL e.g on chromosome 5A (5A1 and 5A2 loci) for the A0132& and on chromosome 1A for the A0022& population. The co-localization of FHB resistance with plant height and maturity QTL could be caused by the contribution of plant height and maturity to disease escape, the pleiotropic effects of FHB resistance genes, or the linkage of the FHB resistance with plant height and maturity genes. In a previous study, after correcting the FHB data for days to heading and plant height, He et al. [47] re-detected a FHB resistance QTL on chromosome 2DL indicating that the resistance to FHB mediated by this locus was not solely due to disease escape. This direct effect on resistance could be the case in the current study, because QTL mapping identified a number of plant height and maturity QTL at loci which were not associated with FHB resistance. Additionally, correlations of plant height and maturity with FHB resistance tended to be low to moderate, suggesting that factors other than just plant height and maturity affect resistance. On the other hand, Steiner et al. [48] indicated that the homologous dwarfing alleles *Rht-B1b* and *Rht-D1b* contribute equally to lower plant height but unequally to FHB susceptibility, supporting the notion that FHB might be modulated by the pleiotropic effect of the plant height genes. Validation of the FHB resistance QTL co-located with the plant height and maturity QTL in other breeding populations with semi-dwarf and early maturing phenotype may more precisely unveil the association between FHB resistance, plant height and maturity.

The understanding of traits associated with a genetic locus is desirable for its utilization in breeding programs. In general, semi-dwarf and early maturing lines are preferred in breeding programs. In this study, FHB resistance and tall or late maturity alleles were detected in the coupling linkage phase. Assuming that the traits are controlled by different tightly-linked genes, the identification of lines that carries favourable alleles for FHB resistance, plant height and maturity would require genetic recombination events to break this linkage. In the case of FHB resistance genes with pleiotropic effects, plant height and maturity could be adjusted using other loci that are not associated with FHB resistance e.g. those identified in this study. Deciding the best strategy for utilization of these loci in the breeding program depends largely on their dissection in future by combining forward and reverse genetic tools.

Several FHB resistance QTL were identified in the A0132& population with 5A1 and 5A2 QTL detected in five different environments and with all the FHB traits analyzed. Phenotyping was initiated with 121 lines of the A0132& population, and continued with 423 lines to evaluate the effect of population size on the detection power of QTL mapping. The minor FHB resistance QTL on chromosomes 1B, 2B and 7A were only detected using the larger set, suggesting the favourable contribution of population size to the detection of these QTL. Population size and precision of phenotypic measurements are two main factors contributing to the detection power of QTL mapping [49]. Li et al. [49] indicated that the population size even outweighs measurement precision. Line DT696 is an adapted durum breeding line and the source of FHB resistance in durum wheat varieties Brigade, Transcend and CDC Credence [32,33]. Improving FHB resistance by utilizing the FHB resistance QTL of line DT696 is promising due to less issue with linkage drag and the moderate heritability of the resistance. Markers identified in this study could be used for stacking the moderate and minor effect FHB resistance alleles of line DT696 to improve the levels of FHB resistance in durum wheat varieties.

QTL mapping identified five FHB resistance QTL in the A0022& population. The QTL on chromosome 1A was detected in five environments with a percentage of phenotypic variance comparable to the FHB resistance QTL on chromosome 3B (*Fhb1*: the largest-effect FHB resistance QTL detected in wheat) in some environments [50,51]. Transfer of *Fhb1* QTL from hexaploid to durum wheat is often confounded by the introduction of undesirable grain quality traits and the weak expression [52]. Transferring the moderate effect FHB resistance QTL on chromosome 1A to durum wheat could have less grain quality penalties than transferring FHB resistance QTL from hexaploid wheat. The moderate effect FHB resistance QTL of a few other tetraploid species such as *T*. *turgidum* ssp. *dicoccoides* [5,19,21] and *T*. *turgidum* ssp. *dicoccum* [6] were successfully transferred into durum wheat, lending support to the feasibility of deploying the FHB resistance QTL of cv. Blackbird. Cultivar Blackbird is an exotic germplasm that has undesirable agronomic traits such as poor straw strength and small seed size. The application of SNP markers associated with the FHB resistance QTL of cv. Blackbird in a marker-assisted backcrossing program should accelerate the utilization of cv. Blackbird resistance. Several strategies are proposed for the application of markers to reduce the effect of linkage drag. For instance, Randhawa et al. [53] could retrieve up to 97% of recurrent parent genome with only two rounds of backcrossing through marker assisted selecting for double-recombinants around the wheat stripe rust gene *Yr15*. We developed a F_2_ population (2500 lines) from a cross between two progenies of the A0022& population with the extreme FHB phenotype and apposite alleles at the FHB resistance QTL on chromosomes 1A, 2B and 6B. Work is underway to apply markers to select the F_2_ double-recombinants for rapid introgression of cv. Blackbird FHB resistance into durum wheat using the method suggested by Randhawa et al. [53].

None of the FHB resistance QTL reported by Somers et al. [8] from Type II phenotyping of the A0022& population under greenhouse conditions were detected under the field conditions. The same discrepancy was reported by Engle et al. [54] when lines with stable resistance in the field failed to express Type II resistance in greenhouse and vice versa. Further support for this environmental effect was observed by Zhang et al. [27] where two FHB resistance QTL on chromosomes 2A and 3A were solely detected under greenhouse condition. These results suggest that the expression of the FHB QTL is modified by the interaction of genotype by environment and that the environment in a greenhouse does not simulate that in the field.

Comparing the position of SSR markers associated with FHB resistance QTL reported by Buerstmayr et al. [29,55] and Buerstmayr and Buerstmayr [46] with SNP markers associated with the FHB resistance QTL detected for the A0132& population on chromosome 5A on the high density tetraploid consensus map [17] suggested that the 5A1 FHB resistance QTL of line DT696 co-located with that from line CM-82036 and the plant height and anther retention QTL from cv. Arina. Previous studies indicated that susceptibility to FHB is lower in winter wheat lines with closed flowers [46]. Plant height and anther retention traits could contribute to disease escape by rendering the microclimate around the spike unfavourable for infection [46]. However, a recent study suggested the direct role of plant height hormone gibberellic acid in resistance of wheat to FHB, lending support to the physiological involvement of plant height genes in resistance to FHB [56]. The association between plant height and spike morphology genes with FHB resistance needs to be elucidated in future studies.

The 5A2 FHB resistance QTL co-located with a major maturity QTL. By comparing the position of markers shared between the map generated in our study and the high-density tetraploid consensus map [17], we found the 5A2 QTL co-located with the FHB resistance QTL detected by Buerstmayr et al. [21] from *T*. *macha* and that QTL detected by Zhang et al. [27] from *T*. *turgidum* ssp. *dicoccum*. Similar comparison with the map generated by He et al. [47] suggested that the vernalisation gene *VRN1* is within the interval. Further mapping studies with larger population size will be needed to decipher the association of the 5A2 FHB resistance QTL with the *VRN1* genes, and the maturity QTL on chromosome 5A.

We determined the FHB resistance QTL on chromosome 1A to be co-located with a maturity QTL. Other research suggests that this is the site of Hessian fly (*Mayetiola destructor*) resistance genes *H9*, *H10*, *H11*, *H16* and *H17* and *Hdic* from *T*. *turgidum* ssp. *dicoccum* [57–59], the powdery mildew resistance gene *Pm3* and the leaf rust resistance gene *Lr10* [59]. Knowing the linkage phase of genes mapped to the distal region of chromosome 1AS in cv. Blackbird is useful for determining the strategy for utilization of this locus in breeding programs. Employing larger mapping populations and the high rate of recombination at the distal regions of chromosomes 1AS may facilitate the decay of the hypothesized unfavourable linkage among genes.

Additive × additive interactions were observed between the FHB resistance QTL identified for both the A0132& and A0022& populations. Pyramiding of FHB resistance QTL additively contributing to resistance is preferred in breeding programs. Theoretically, the levels of resistance could be boosted in lines carrying FHB resistance alleles with additive × additive interactions. Ma et al. [60] also found additive × additive interaction among nine pairs of loci mediating FHB resistance in the Chinese Spring-Sumai 3 chromosome 7A disomic substitution lines. The QTL interaction analysis found loci on chromosomes 4A, 4B, 5A and 7A interacting with the FHB resistance QTL of cv. Blackbird on chromosome 1A, none of which was identified as significant QTL using the MQM analysis. McCartney et al. [61] also found four digenic interacting FHB loci, which were not identified using a one-dimensional scanning method. Interestingly, the locus on chromosome 5A interacting with the FHB resistance QTL on chromosome 1A co-located with the 5A2 FHB resistance QTL identified for the A0132& population. The additive × additive interaction of the 5A2 locus with the FHB resistance QTL on chromosome 1A along with its association with the moderate effect FHB resistance QTL of line DT696 and several domestication-related genes suggests the complex role of this locus in the phenotypic expression of FHB resistance in tetraploid wheat.

## Conclusions

In conclusion, the phenotypic expression of FHB resistance in the A0022& and A0132& populations was partially impacted by environment. QTL mapping identified multiple loci conferring FHB resistance in the populations. The detected loci varied in consistency of expression over environments, with the FHB QTL on chromosome 1A derived from cv. Blackbird and those on chromosome 5A derived from line DT696 being more consistently expressed. Assembly of the FHB resistance QTL on chromosomes 1A and 5A into a new durum wheat variety could be a sound strategy for lowering the susceptibility of durum wheat to FHB. However, the co-localization of these QTL with plant height and maturity QTL impose some challenges for their utilization. These challenges could be tackled by selection for dwarfing and early maturity alleles at the loci associated with plant height and maturity but not-associated with FHB resistance. The utilization of FHB resistance QTL on chromosome 1A requires reducing the effect of linkage drag through backcrossing. The application of SNP markers will improve the throughput and speed of marker-assisted backcrossing for introgression of cv. Blackbird resistance into durum wheat. The additive × additive interaction among the minor and moderate effect FHB resistance QTL identified here promises improvement in the levels of resistance in lines carrying a combination of resistance alleles. Identification of the FHB resistance QTL conferring resistance in line DT696 and cv. Blackbird and the associated SNP markers contributes to the speed and precision of gene pyramiding and marker-assisted backcrossing programs and could pave the way for improving FHB resistance in durum wheat.

Work is underway to validate these markers by phenotyping and genotyping of over 1000 lines of the SCRDC-AAFC durum breeding program. Fine mapping of the FHB resistance QTL on chromosomes 1A and 5A is underway and could lead to the identification of more reliable markers and the higher precision of marker-assisted selection.

## Supporting information

S1 Table Summary of field nursery locations and years, number of replicates, population size and inoculation methods used to phenotype the DT707 × DT696 (A0132&) and the Strongfield × Blackbird (A0022&) populations for Fusarium head blight (FHB), plant height and maturity. Location abbreviations: *Portage la Prairie, †Carman, ‡Morden, **Brandon, ǂRegina, §Lethbridge, ††Swift Current, §§Indian Head.
(DOCX)

S1 File High density genetic map and, results of QTL mapping and QTL interaction analyses for the A0132& population. (XLSX)

S2 File High density genetic map and, results of QTL mapping and QTL interaction analyses for the A0022& population. (XLSX)
