# Supplementary material for: High density genetic mapping of Fusarium head blight resistance QTL in tetraploid wheat
Source: PLoS One. 2018 Oct 11;13(10):e0204362. doi: 10.1371/journal.pone.0204362 (PMC6181299; doi:10.1371/journal.pone.0204362)
Supplement: S1 Table — Location abbreviations: *Portage la Prairie, †Carman, ‡Morden, **Brandon, ǂRegina, §Lethbridge, ††Swift Current, §§Indian Head. (DOCX) [file pone.0204362.s001.docx]

| Population | Traits | Population size | Location | Year | Number of replicates | Inoculation method |
| --- | --- | --- | --- | --- | --- | --- |
| A0132& | FHB | 121 | PLP* | 2006 | 2 | Corn Spawn |
|  |  |  |  | 2007 | 2 |  |
|  |  |  | CAR† | 2005 | 2 | Conidial spray |
|  |  |  |  | 2006 | 2 |  |
|  |  |  |  | 2007 | 2 |  |
|  |  | 423 | MD‡ | 2015 | 1 | Corn Spawn |
|  |  |  |  | 2016 | 1 |  |
|  |  |  |  | 2017 | 1 |  |
|  | Plant height and maturity | 121 | LB§ | 2004 | 1 |  |
|  |  |  | Regǂ | 2005 | 2 |  |
|  |  |  |  | 2006 | 2 |  |
|  |  |  |  | 2007 | 2 |  |
|  |  |  | SC†† | 2004 | 2 |  |
|  |  |  |  | 2005 | 2 |  |
|  |  |  |  | 2006 | 2 |  |
|  |  |  |  | 2007 | 2 |  |
|  |  |  |  | 2015 | 2 |  |
| A0022& | FHB | 90 | PLP | 2006 | 2 | Corn Spawn |
|  |  |  |  | 2007 | 2 |  |
|  |  |  | CAR | 2006 | 2 | Conidial spray |
|  |  |  |  | 2007 | 2 |  |
|  |  | 102 | MD | 2015 | 1 | Corn Spawn |
|  |  |  |  | 2016 | 1 |  |
|  |  |  |  | 2017 | 1 |  |
|  |  |  | IH§§ | 2015 | 1 | Natural infection |
|  |  |  | BD** | 2016 | 1 | Corn Spawn |
|  |  |  |  | 2017 | 1 |  |
|  | Plant height and maturity | 90 | Reg | 2005 | 2 |  |
|  |  |  |  | 2006 | 2 |  |
|  |  |  | SC | 2005 | 2 |  |
|  |  |  |  | 2006 | 2 |  |
|  |  |  |  | 2012 | 2 |  |
|  |  |  |  | 2014 | 1 |  |
